# Spinal Epidural Lipomatosis Causing Lumbar Canal Stenosis: A Pictorial Essay on Radiological Grading and the Role of Bariatric Surgery Versus Laminectomy

**DOI:** 10.7759/cureus.26492

**Published:** 2022-07-01

**Authors:** Sunil Manjila, Michael Fana, Khalid Medani, Matthew D Kay, Rehan Manjila, Timothy G Bazil, Unni Udayasankar

**Affiliations:** 1 Neurosurgery, McLaren Bay Region Medical Center, Bay City, USA; 2 Neurology, Central Michigan University College of Medicine, Saginaw, USA; 3 Occupational Medicine, Loma Linda University Medical Center, Loma Linda, USA; 4 Radiology, University of Arizona College of Medicine, Tucson, USA; 5 Swanson School of Engineering, University of Pittsburgh, Pittsburgh, USA; 6 Physical Medicine and Rehabilitation, Beaumont Hospital, Royal Oak, USA; 7 Medical Imaging, College of Medicine, University of Arizona, Tucson, USA

**Keywords:** laminectomy, bariatric surgery, mediastinal lipomatosis, obesity, epidural lipomatosis

## Abstract

Spinal epidural lipomatosis (SEL) is a rare condition characterized by an excessive accumulation of adipose tissue in the spinal canal that can have a compressive effect on intraspinal neuroanatomical structures, leading to clinical symptoms. Several different conservative and surgical treatment strategies have been proposed but the treatment and outcomes remain controversial. There is a lack of severity-based evidence documenting the success of decompressive laminectomy in SEL and there are only anecdotal reports of clinico-radiological success with weight loss from bariatric surgery. This article demonstrates the resolution of SEL in two patients with bariatric surgery with the help of pre and postoperative MR imaging. The authors also highlight the classic “types” of spinal epidural lipomatosis with a surgically relevant grading system and elucidate the existence of concurrent extraspinal lipomatosis (i.e. mediastinal and intra-abdominal lipomatosis), drawing parallels with the natural history of SEL. The controversial question remains whether a symptomatic SEL patient needs a multilevel laminectomy for spinal decompression or bariatric surgery that can indirectly help the spinal condition. We propose that bariatric intervention could be better frontline management in patients with multifocal/multisystem lipomatosis (i.e., combined spinal and extraspinal) and spinal decompression would be ideal for those SEL patients with coexisting bony and/or ligamentous spinal canal or foraminal stenosis. This manuscript serves as a comprehensive and contemporary update on the radiological profile and two plausible treatment paths and will look toward further verification by a randomized clinical trial.

## Introduction and background

Spinal epidural lipomatosis (SEL) is a benign condition characterized by epidural accumulation of excessive non-encapsulated adipose tissue that distorts or compresses the thecal sac and may cause lower back pain, neurogenic claudication, acute or progressive paraparesis, cauda equina syndrome, and radiculopathy, depending on the location and extent of stenosis [1-11]. There is a demonstrated male predominance, and an overall prevalence of 2.5% that can be classified into those who are asymptomatic (0.6%), symptomatic (1.8%), or symptomatic attributable to SEL (0.1%) [12-14]. According to Malone et al., the prevalence of SEL in patients with spinal stenosis is 6.26% and the incidence of severity according to grades 2 and 3 Borre criteria is 1.54% (Borre series 4.1%) and 0.96% (as against Borre’s series citing 2.2%), respectively [13,15].

Patients present with SEL in the lumbar spine more than the thoracic spine. Patients who develop the thoracic variant of SEL may present with painless, slowly progressive myelopathy. Similarly, those with a lumbar lesion typically present with back pain with or without slowly progressive lower extremity weakness. This may progress to an acute paraparesis due to coexisting vertebral osteoporotic fractures from chronic steroid use [16]. Sensory symptoms (e.g., tingling and numbness) are reported along with rare occurrences of sphincter disturbances with a cauda equina presentation. Rapid neurologic deterioration in SEL and delayed occurrence of focal SEL after a single spinal steroid injection have been reported [17-18].

Various etiologies have been reported for SEL, including exogenous steroids, endogenous steroid disease, obesity, and idiopathic conditions [2,4-5,19-24]. Other associations proposed include abnormalities in spinal curvature (e.g., scoliosis, kyphosis, and Scheuermann’s disease), HIV treatment with highly active antiretroviral therapy (HAART), endocrine disorders (e.g., prolactinoma and Cushing’s syndrome), metabolic bone disorders (e.g., fluorosis, Paget’s disease, and tumoral calcinosis), and hypothyroidism [25-34]. We further postulate that hypoplasia of the L5 vertebra may be an associated risk factor for lumbar SEL. Originally described as pseudospondylolisthesis by Frank and Miller (1979), Wilms et al. (2008) examined 2223 patients with a degenerative lumbar disease and discovered 22 patients with hypoplasia of the L5 vertebra [35-36]. Subsequent investigations have reported similar findings with significant posterior wedging of the L5 vertebra [37-38]. Moreover, Bagheri et al.’s examination of patients undergoing lumbosacral MRI for various conditions found 2% of this population with pseudolisthesis secondary to L5 hypoplasia [38]. Other radiographic features that may suggest L5 hypoplasia include focal widening of the spinal canal, presence of hypoplastic pedicles and facets, disc desiccation at the L4-L5 and L5-S1 levels as associated with pseudodisc bulges in the inferior and superior directions, respectively [36]. Therefore, it is possible that hypoplasia of the L5 vertebral body with focal canal widening may make SEL relatively asymptomatic for a more prolonged time than the more expansive growth of SEL rostrocaudally, which would lead to early symptomatic presentation.

As of today, there is a lack of documented success of decompressive laminectomy in SEL correlated with symptom severity, and only limited reports of clinico-radiological success in SEL are available with weight loss from bariatric surgery. Here, we demonstrate the resolution of SEL in two patients with prior bariatric surgery, validated by pre and postoperative MR imaging. We further aim to classify the more common types of SEL with a surgically relevant grading system and elucidate the existence of concurrent extraspinal lipomatosis (i.e. mediastinal and intra-abdominal lipomatosis), drawing parallels with the natural history of SEL.

## Review

Methods

This narrative review was prepared using the Preferred Reporting Items for Systematic Reviews and Meta-Analyses (PRISMA) statement. The MEDLINE database (via PubMed) was searched using the following search term: “epidural lipomatosis” with no time limit set. The search was then narrowed by selecting the “human” link for species and the “English” link for language. The article titles and abstracts were reviewed for relevance and duplications. Select articles were then included in the final review. Articles that contained information relevant to the pathophysiology, epidemiology, diagnosis, clinical implications, and therapeutic interventions for epidural lipomatosis were included and organized according to these categories. The individual articles were accessed and pertinent information according to each category was extracted for comparison. Our search resulted in 299 publications that were narrowed to 185 articles based on the inclusion criteria and relevance to epidural lipomatosis.

Of the 185 articles reviewed, 128 were case reports, with the remaining 57 comprising case series or reviews that also assessed causes or associations to help with understanding the potential pathophysiology, epidemiology, diagnosis (primarily reliant on imaging modalities), clinical implications, and therapeutic interventions. For our final cross-analysis of case reports documented thus far, we include 12 reports of SEL demonstrating clinical (BMI) and radiological (MRI) improvement with either a weight loss program or sleeve gastrectomy, as well as two of our own retrospective patient cases (Supplementary Image 1).

Imaging in SEL

SEL is a non-encapsulated and benign intraspinal lesion involving the narrowing of the spinal canal. While it may appear similar to intraspinal extradural lipomas, the latter are benign tumors seen as a well-demarcated focal mass on imaging, composed of mature lipocytes, and enclosed by a fibrous capsule frequently associated with spinal dysraphism [39]. Moreover, if abnormal vascular elements are seen interspersed amongst the mature lipocytes with local infiltration, a differential diagnosis of spinal epidural angiolipoma is entertained, especially in the setting of evidence of post-contrast enhancement [40].

**Figure 1:**
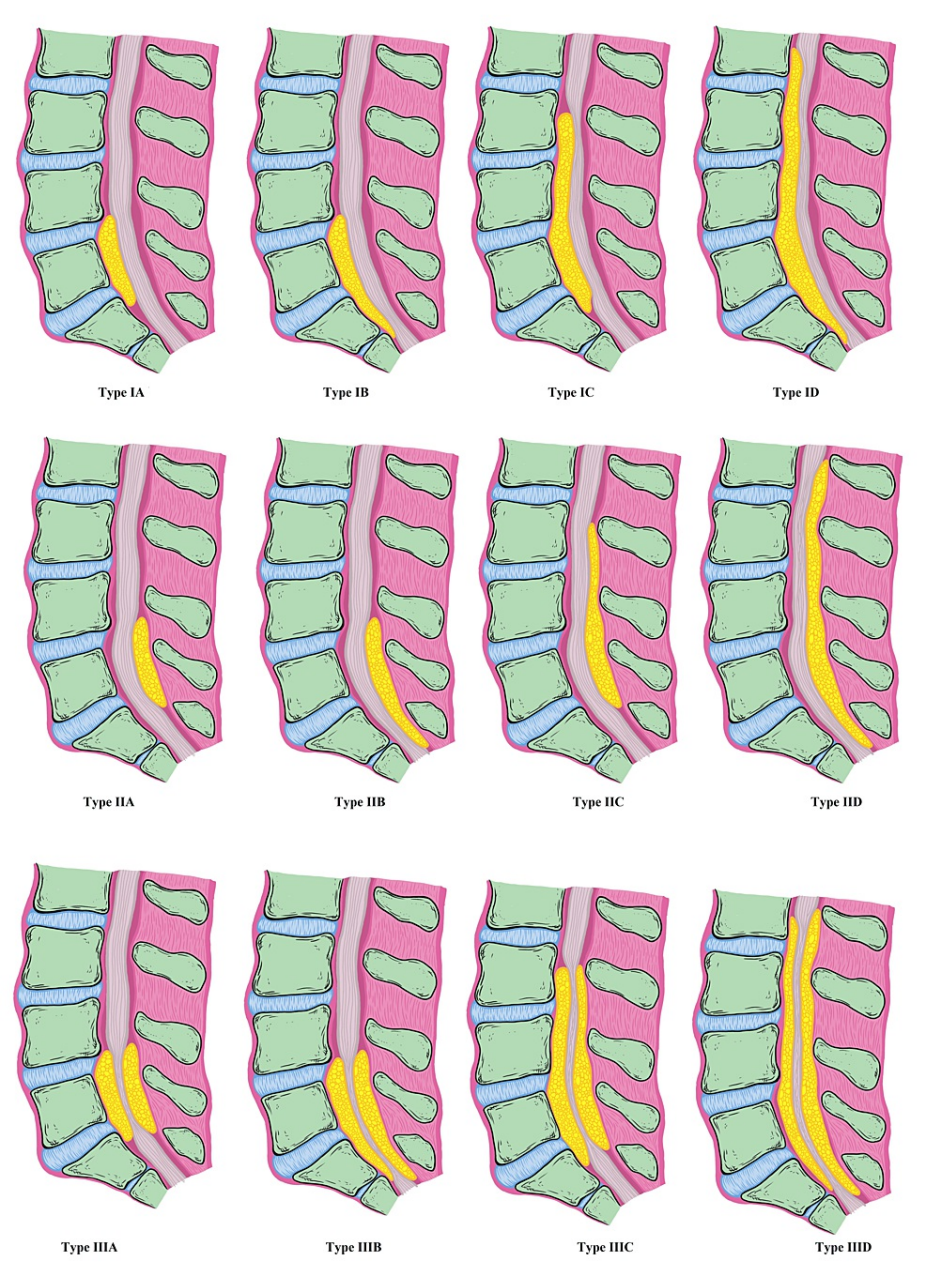
Artistic rendition of Manjila types I-III SEL classification: ventral, dorsal, and circumferential types called types I, II, and III, respectively Sagittal views of these three types show four subtypes A-D. An L5 body with or without apparent wedging or pseudospondylolisthesis is considered a reference point. Subtype A: SEL restricted to the level or height of the L5 body. Subtype B: SEL extending caudal to the level of the L5 body. Subtype C: SEL extending rostral to the level of the L5 body. Subtype D: SEL extending both rostral and caudal to the level of the L5 body. This novel classification can be used for effective communication among medical providers and follow-up of post-procedure SEL status after a weight loss program or surgical intervention. SEL: spinal epidural lipomatosis Source: Authors of the current article

While SEL may present at any location along the vertebral column, it is rare in the cervical spine with only anecdotal case reports in the literature [41]. Lumbar, followed by thoracic-level, SELs are more common. The classic radiological patterns of SEL that the authors propose here are as follows: ventral (anterior; Manjila classification Type I), dorsal (posterior; Manjila classification Type II), and concentric (circumferential or napkin-ring pattern; Manjila classification Type III) (Figure 1). Each type is further subtyped as A, B, C, and D as depicted in Figure 1. For instance, subtype A in type I SEL is a restricted SEL behind an L5 vertebral body; B extends caudally from the L5 level; C extends rostrally from the L5 level; D extends both rostrally and caudally from the L5 level (Figure 1). Additionally, the authors subcategorized each type into Alpha (α), Beta (β), and Gamma (Subcategory Alpha (α) is associated with spinal compressive conditions (such as synovial cysts, facet hypertrophy, disc-osteophyte complexes, etc.). Subcategory Beta (β) is associated with extraspinal lipomatosis (e.g., subcutaneous, mediastinal, and visceral lipomatosis). Subcategory Gamma (γ) is associated with both conditions in Alpha and Beta (Table 1).

**Table 1:** Manjila classification of SEL showing both extent and severity of the disease using sagittal and axial MRI sequences. Note: Subcategory Alpha (α) depicts coexisting bony/ ligamentous/ synovial degenerative processes. Subcategory Beta (β) denotes the presence of extraspinal lipomatosis (subcutaneous/ mediastinal/ visceral). Subcategory Gamma (γ) comprises a combination of both Alpha and Beta. SEL: spinal epidural lipomatosis

Location (Spinal)	Type	Subtype (Based on Sagittal MRI)	Grade (Based on Axial MRI)
Eccentric	Type I (Ventral)	A – SEL restricted to the level or height of the L5 body (ventral to the thecal sac)	Mild, Moderate, or Severe
		B – SEL extended caudally from Type A	
		C – SEL extended rostrally from Type A	
		D – SEL extended both rostrally and caudally from Type A	
	Type II (Dorsal)	A – SEL restricted to the level or height of the L5 body (dorsal to the thecal sac)	Mild, Moderate, or Severe
		B – SEL extended caudally from Type A	
		C – SEL extended rostrally from Type A	
		D – SEL extended both rostrally and caudally from Type A	
Concentric	Type III (Circumferential)	A – SEL restricted to the level or height of the L5 body (in a circumferential manner)	Mild, Moderate, or Severe
		B – SEL extended caudally from Type A	
		C – SEL extended rostrally from Type A	
		D – SEL extended both rostrally and caudally from Type A	

Even though the clinical findings on physical exam are rather non-localizing, multiplanar spinal imaging is cardinal for grading the severity of SEL [14,42-43] The neurogenic claudication associated with uncomplicated SEL is typically not as profound as those with spondylotic canal stenosis. There are usually no definitive radiculopathies seen on either neurological examination or electromyography (EMG), leaving the decision for possible intervention to the grading of SEL on imaging studies. CT imaging in SEL will show a focal epidural filling defect with smooth margins and the epidural space having adipose tissue density ranging from -80 to -120 HU, distinguishing it from non-fat tissues. There will be variable flattening of the thecal sac as evidence of a focal mass effect dorsally or ventrally. Myelography, on the other hand, would show a typical hour-glass pattern of concentric or circumferential obstruction and demonstrate whether the lesion is intradural or extradural.

The MRI diagnosis of SEL requires demonstration of an increased amount of fat within the epidural space, homogenous in intensity, without transition boundaries, and well-demonstrated on T1, T2, and fat-suppression sequences but with variable mass effect on the thecal sac [12].

**Figure 2:**
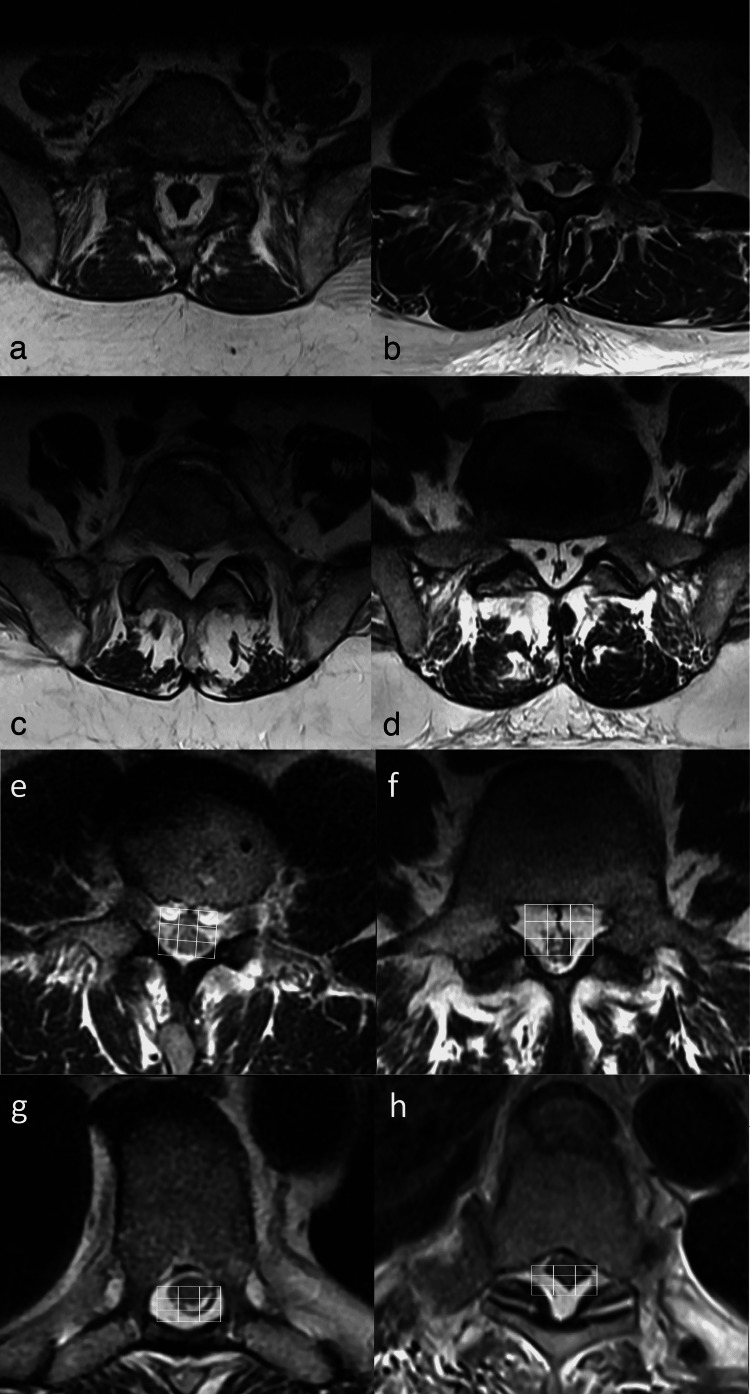
a-d: Axial T2-weighted MR images demonstrating different thecal sac morphologies in Manjila Type III lumbar SEL a) polygonal, b) stellate, c) Y-shaped and d) nearly obliterated thecal sac. Oftentimes, the radiological severity does not translate to the clinical severity of radiculomyelopathy or neurogenic claudication. e-f: Axial T2-weighted MR images demonstrating a novel 3x3 grid method in e) mild type Manjila Type I (ventral) and f) moderate Manjila Type I (ventral) SEL cases. g-h: depict a thoracic SEL Manjila Type II (dorsal) variant, where a 3x3 grid can be readily applied, as against a 3+1 or trifoliate grid required at the L5-S1 level. SEL: spinal epidural lipomatosis Source: Authors of the current article

We have also encountered various shapes of compressed thecal sac on axial MRI sections that range from polygonal, stellate, and Y-shaped, to near-complete obliteration (Figures 2a-2d). Likewise, Borre et al. (2003) report patients with lumbosacral SEL and flattening of the thecal sac in those with thoracic SEL [15]. The classic “Y” sign on axial MR images at the lower lumbar and sacral levels is considered a hallmark of severe concentric stenosis and a more typical anteroposterior dural sac flattening in Manjila Types I and II SEL, especially in the thoracic spine [44].

We have noted more detailed, but rather non-specific, intraspinal anatomy in the MR imaging for SEL, ranging from a ventral-predominant orientation of the epidural venous plexus to the extent of anchoring meningo-vertebral ligaments that extend from the ventral aspect of the thecal sac to the osseous or capsular structures at the inner margins of the bony central canal [45]. Although epidural veins and meningo-vertebral ligaments are variable in number and location, both appear to be more anterior to the thecal sac than posterior. With the increasing severity of stenosis, however, the epidural veins become less prominent and disappear as the meningo-vertebral ligament becomes profoundly visible ventral to the thecal sac, especially in severe cases of Manjila Type I and Type III SEL.

In addition to offering a clear radiological differentiation between SEL, epidural angiolipoma, and intraspinal epidural lipoma, the MR images can provide information regarding the presence of obesity variants: subcutaneous, mediastinal, and visceral (intraabdominal) [46]. The visceral type of obesity has been shown to have a strong association with increased risks for cardiovascular disease, diabetes, and metabolic syndrome. Identification of SEL at earlier stages of obesity or extraspinal lipomatosis (Manjila subcategory Beta for extraspinal lipomatosis) can help initiate early weight loss and physical therapy plans to prevent the development of symptomatic SEL.

Discussion

Existing Radiological Grading of SEL

The initial grading system proposed by Borre et al. (2003) was also based on MRI due to its well-known multiplanar nature, lack of ionizing radiation, and superior soft-tissue contrast delineating the outline of the thecal sac, fat, and intradural structures [15]. This sharp soft tissue contrast differentiates epidural fat from adjacent ligaments (i.e. ligamentum flavum) and cerebrospinal fluid that surrounds the spinal cord or cauda equina. The grading scale (0 to III) is based on the ratio or percentage of the spinal canal diameter that is occupied by the epidural fat. Grade I ranges from 41% to 50%; grade II from 51% to 74%; and grade III equal to or greater than 75%. Notably, all SEL patients with Borre grade III epidural lipomatosis had symptoms [15,43]. Their grading system was initially demonstrated in the lumbar spine but has subsequently been correlated in the other spine regions.

However, the Borre grading system has the disadvantages inherent to using cross-sectional computing. In patients with severe lumbosacral SEL, where stenosis ranges from polygonal, stellate, and Y-shaped to nearly complete obliteration, it is difficult to delineate dural outlines, especially in areas of square centimeters. Similarly, when the axial MRI slices are originally acquired, it is cumbersome to calculate the area with adjustment for associated facet pathology (e.g. large or asymmetric synovial cysts or facet hypertrophy) or vertebral malalignment, as in scoliosis. This classification is not reliable as a guideline for planning a definitive surgical approach or reflecting the postoperative clinical outcome. Hence, the authors propose a new classification that can explain both the extent and severity of SEL.

Other grading systems include subjective estimations of the total epidural fat surface area with mild SEL indicating spread along to the surface area of the dural sac, moderate SEL comprising twice the area of the dural sac, and severe SEL enveloping more than twice the area of the dural sac [47]. Some imaging experts consider the epidural adipose tissue with an anteroposterior thickness of 7 mm or greater as a diagnostic criterion for SEL based on the inner diameter of the bony spinal canal used as outer reference points. With the abovementioned grading systems, it is difficult to differentiate between mild SEL and normal epidural fat on MR imaging of the spine. Likewise, computing the degree of SEL at the L5-S1 level versus the rest of the spine by comparison of cross-sectional areas can be erroneous given the trifoliate anatomy of the former. Hence, a large retrospective study would need to be performed to obtain normative data for SEL along various regions of the spine and compare cross-sectional versus grid systems for the classification of SEL. Of special mention is the extent and severity of concurrent ligamentous stenosis where the mid-laminar measurement is employed to assess ligamentum flavum hypertrophy leading to 'radiologic' stenosis.

Proposal of a Novel SEL Classification and Grading System

**Figure 3:**
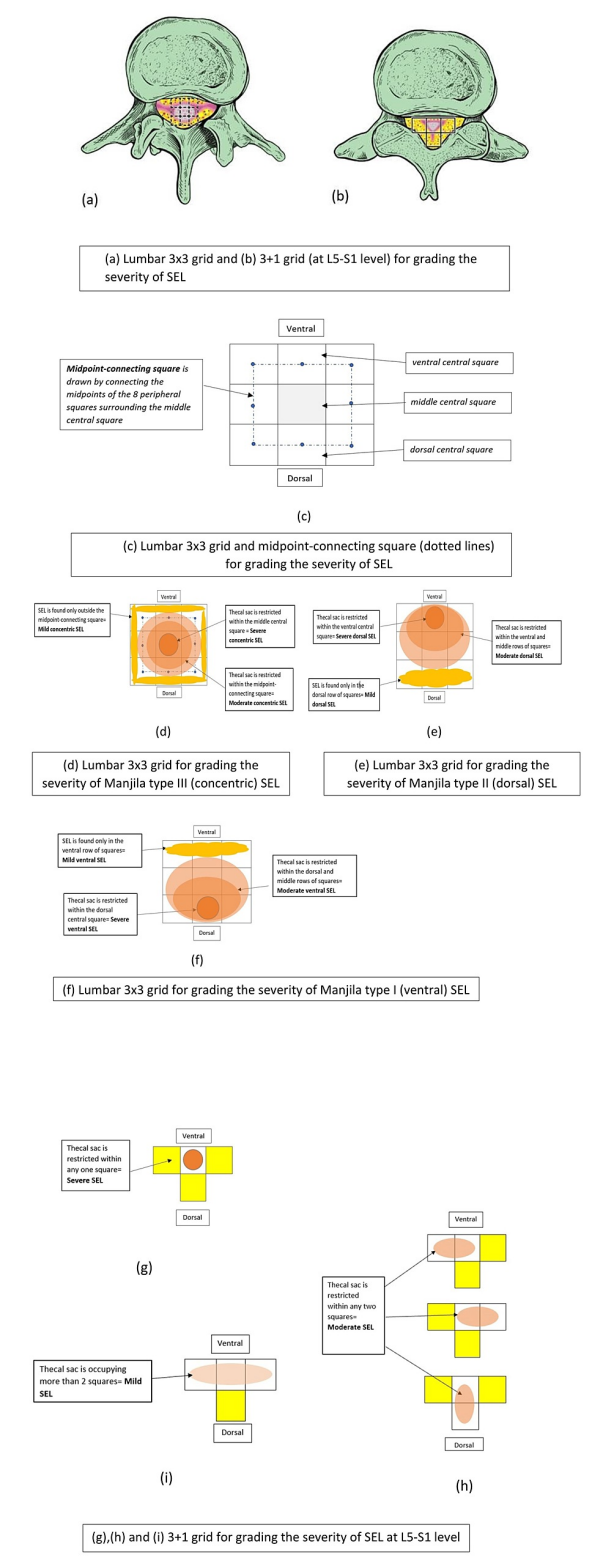
Illustration of the grading of the severity of SEL using the a) 3x3 grid method, and b) 3+1 grid (used exclusively for the L5-S1 levels, given the typical trifoliate pattern of the spinal canal). c) and d) are a diagrammatic representation of the 3x3 grid in the concentric SEL where the midpoint-connecting square is drawn to assess its severity. e) and f) show the use of the 3x3 grid in grading the dorsal and ventral SEL respectively. g)-i) show the use of the 3+1 grid at the L5-S1 level. g) shows severe SEL where the thecal sac is in any one square. h) shows moderate SEL where the thecal sac is occupying two squares (axial or sagittal). i) shows mild SEL where the thecal sac is occupying more than two squares. While the sagittal MRI is used to define the type of SEL (concentric, dorsal, and ventral), the axial MRI with maximum thecal compression is used for grading the severity. This novel composite grading offers easy adaptability and reproducibility among both medical and paramedical personnel. SEL: spinal epidural lipomatosis Source: Authors of the current article

We propose a novel and simple classification system that is intuitive and user-friendly to calculate the severity of the thecal compression in SEL. This classification system utilizes a 3x3 grid (Figures 3a, 3c-3f) manually drawn or annotated on axial spinal imaging and interpreted by the radiologist or spine surgeon. This can be done even in patients with associated canal stenosis or scoliosis without using additional computing software for relative cross-sectional areas that typically use a cm^2^ area function. Furthermore, this classification is surgically relevant to the operative corridors of access and the feasibility of decompressing the thecal sac based on the epicenter of the SEL. This system can be effectively used to assess the progression of SEL in large-volume studies and screen for severe (i.e. surgical) SEL cases. This novel grid technique is easier to perform and get familiarized or disseminated compared to drawing cross-sectional areas in complex axial SEL morphologies, especially in Y- or stellate-pattern SEL or those at the trifoliate L5-S1 level. This novel MRI-based classification offers easy adaptability and reproducibility for medical providers and can be used for prognostication and follow-up comparison studies. This template of Manjila classification can also be employed in machine learning algorithms in radiological grading of this condition, rendering it easily interpretable by artificial intelligence solutions.

An exception to the 3x3 grid rule would be a lesion causing stenosis at the L5-S1 level where the spinal canal has a trifoliate morphology. Here, a 3+1 grid system is used where the lower grid corners are placed arbitrarily at the medial end of the facet joint line (Figures 3b, 3g-3i).

**Figure 4:**
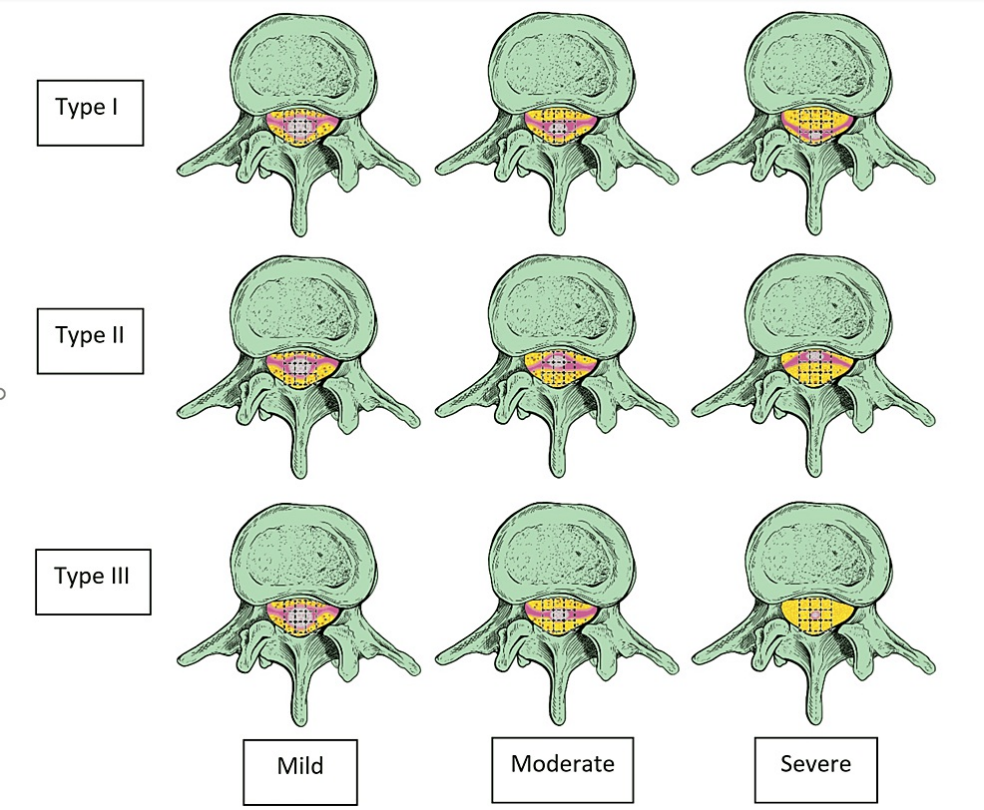
Schematic representation of the Manjila classification of SEL Artistic rendition of the mild, moderate, and severe grades of Types I, II, and III Manjila classification of SEL. SEL: spinal epidural lipomatosis Source: Authors of the current article

As a general rule in our novel classification system, the extent of the SEL lesion is assessed on the sagittal view MRI while the coronal MRI views are not used for classification. Axial views of the spinal MRI are used to assess the severity of stenosis in SEL. For eccentric-type SEL (Manjila Types I and II); mild SEL is defined as epidural fat occupying only some or all of the three ventral squares (Manjila Type I) or some or all of the three dorsal squares (Manjila Type II). Moderate SEL is defined as thecal sac being restricted to either middle and dorsal squares (Manjila Type I) or middle and ventral squares (Manjila Type II). Severe SEL is defined as thecal sac being restricted to either the dorsal middle square (Manjila Type I) or the ventral middle square (Manjila Type II) (Figures 3e, 3f, and Figure 4).

To determine the severity of the concentric-type SEL (Manjila Type III), a midpoint-connecting square is drawn by connecting the midpoint of each peripheral square surrounding the middle central square (Figure 3c). In this case, mild SEL is defined as epidural fat located outside the midpoint-connecting square, while moderate SEL is defined as thecal sac is restricted inside the midpoint-connecting square, and severe SEL is defined as the thecal sac being restricted to the middle central square (Figure 3d and Figure 4). At the L5-S1 level, using the 3+1 grid, mild SEL is defined as thecal sac is occupying more than two squares while moderate SEL is defined as the thecal sac being restricted to two squares, and severe SEL is defined as the thecal sac being restricted to one square (Figure 3g-3i).

With an additional screening of the chest and abdomen, multisystem staging of lipomatosis can be done and early weight loss programs can be instituted in patients with SEL. It must also be noted that SEL patients can have multilevel canal stenosis of varying degrees, either from the lipomatosis itself or coexisting bony or ligamentous stenosis (as in subcategory Alpha). Therefore, it is important to grade the patient’s multilevel disease process (through the subcategories Alpha, Beta, and Gamma mentioned earlier).

Obesity and Metabolic Syndrome

In 2016, the prevalence of obesity in the United States approximates 40% and affects over 93 million adults [48]. Borre and Sugaya respectively report that 87% and 60% of their documented SEL patients were obese. Additionally, prior studies demonstrate a correlation between SEL and obesity, BMI, diabetes mellitus, and metabolic syndrome. For example, a BMI greater than 28 demonstrates a linear relationship with the incidence of SEL [13,47,49]. Other studies demonstrate a correlation between SEL and increased abdominal circumference and visceral fat (but not the total fat percentage or subcutaneous fat) as well as elevated levels of insulin, uric acid, ferritin, inflammatory cytokines (e.g., tumor necrosis factor-alpha (TNF-α) and interleukin (IL)-1β) and triglycerides [47,50-51]. It is possible that adipokines such as leptin and adiponectin play a regulatory role, as it is implicated in hepatic steatosis, which may also occur in metabolic syndrome [50,52-54].

Role of Steroids in SEL: A Faustian Dilemma With Epidural Injections

Lee et al. (1975) reported the first case of steroid-induced SEL after a renal transplant in 1975 [22]. Similarly, Buthiau et al. (1988) also reported epidural fat expansion on follow-up CT scans after one year of systemic steroid use, confirming the presence of SEL as a clinical and radiological entity in 1988 [55]. In fact, the youngest patient with SEL reported in the literature is a six-year-old male with a history of steroid treatment diagnosed with “epidural hibernoma.” Many case reports and series followed, showing the link between steroid usage and SEL as one of causation rather than association in both young and adult patients [56-57]. Al-Khwaja reported 111 patients with SEL with over 50% reported to use exogenous steroids, now known as the most significant medical risk factor of SEL [2]. Duration and dosages of steroids used in these patients who developed SEL appeared to vary (mean daily doses of prednisone range from 30 to 100 mg with a mean duration of 5-11 years) and this does not preclude inhaled forms. Lower doses and shorter durations of steroids have also been reported [12].

Not surprisingly, epidural steroid injections are generally contraindicated in established cases of SEL [58-59]. Malone et al. (2018) reported 17 patients with epidural steroid injections prior to a diagnosis of SEL, seven of whom had Borre grade 3 SEL while the remaining 10 with grade 2 [13]. Whether those patients with SEL can get epidural or facetal injections of steroids for concurrent symptomatic spinal pathologies seems to be a Faustian dilemma [60]. Some interventional pain physicians believe that epidural steroid injections are an absolute contraindication in SEL because they may fail to provide analgesia due to epidural fat filling the spinal canal and instead accentuate epidural fat deposition, worsening the pain and neurological symptoms. Some other clinicians believe there is more water content in epidural fat which can alter the bioavailability of the injected steroids [61]. Nonetheless, more randomized clinical and radiological studies must be performed to validate either hypothesis.

Extraspinal Multisystem Lipomatosis

Extraspinal lipomatosis can occur in the chest, abdomen, pelvis, and extremities apart from axial subcutaneous locations [62]. Mediastinal lipomatosis is a relatively benign condition caused by the deposition of mature adipose tissue in the mediastinum that results in widening, especially of the upper mediastinum (Supplementary Image 2) [63]. In certain cases, a progression in mediastinal lipomatosis is shown with increasing BMI (Supplementary Image 3). Subcutaneous lipomatosis is another common extraspinal condition with a higher chance of recurrence after lipolysis [62]. Madelung disease, also known as multiple symmetric lipomatosis (MSL), involves symmetric deposits of unencapsulated fat in regions of the upper neck, torso, abdomen, and proximal limbs [64]. In patients with visceral lipomatosis, typically omental lipomatosis (Supplementary Image 4), there is an increased incidence of diabetes mellitus, cardiovascular disease, and metabolic syndrome.

Treatment of extraspinal lipomatosis typically involves surgical excision or decompression (i.e., tumescent liposuction, lipectomy, or injection lipolysis). Removal of mediastinal or omental lipomatosis may be mandated only by rare secondary aerodigestive compression symptoms. Subcutaneous lipomatosis lesions are typically removed for pain control or aesthetic reasons.

Management of SEL

Conservative management of SEL includes reduction or cessation of exogenous steroids if applicable and enrollment in a supervised weight loss program. Weight loss is not limited to dietary or physical activity modification but also includes bariatric surgical procedures [51]. However, since bariatric surgery requires a great deal of planning and patient preparation, including optimization of systemic medical comorbidities as well as psychological counseling, SEL has been managed primarily with conservative medical management.

Role of Spinal Decompression Surgery in SEL

Decompressive laminectomies are the mainstay of treatment in symptomatic SEL [65-67]. Some case reports have mentioned extensive fat-debulking as an additional procedure to a standard laminectomy. Surprisingly, a preoperative low dose of steroids is reported to improve chances for better neurological recovery [55]. Moreover, many patients with idiopathic SEL tend to have a better clinical prognosis with complete recovery after surgical decompression [12]. In some anecdotal cases, patients require multiple surgeries because of re-accumulation of epidural fat [12].

Minimally invasive endoscopic procedures or more extensive surgical decompression techniques are selected in the surgical management of SEL based on the surgeon’s preference or expertise [20,33-34,43,68]. Debulking of epidural adipose tissue has been performed using hooks, disc punches and curettes, suction, and bipolar cautery.

Based on our classification system, Manjila Type I SEL almost never gets treated by direct adipolysis as a posterior decompression would indirectly produce the desired surgical outcome. A direct surgical approach to this ventral intraspinal lesion would require a cumbersome anterior or posterolateral access. Manjila Types II and III would be amenable to conventional laminectomy and direct fat excision or lipolysis for decompression of the thecal sac because the epicenter of the lesion is either entirely or partially, respectively, located posterior to the thecal sac.

Even though adipose tissue can be removed by suction or scraping through a limited laminotomy, a conventional wide decompressive laminectomy is frequently employed to treat SEL. Concurrent skipped laminectomies may also be performed for treating tandem thoracolumbar lesions that cause synchronous mass effects at two discrete points on the spinal neuraxis. There are technical limitations to removing long-segment rostrocaudal extents of SEL through laminectomy or laminotomy since there are no flexible debriders that can be safely used far under the laminae rostrocaudally for lipolysis. Hence, surgeons resort to regular suction instruments or catheters for SEL removal. There is an inherent risk for postoperative acute or delayed epidural hematoma formation in the space created by removed adipose tissue, which is akin to intradural spine surgeries where the CSF egress would collapse the thecal sac.

Similarly, in Manjila Type III (severe) concentric SEL, lipolysis offers access to almost 270 degrees axially around the thecal sac. However, the suction of fat should be performed quickly as the rapid expansion of the erstwhile compressed thecal sac into the surgical field can completely impede the view of the ventral fat. Nonetheless, our clinical experience shows that patients have good symptomatic relief even with the removal of the dorsal part of the circumferential SEL. A case report demonstrating the successful use of an epidural spinal cord stimulator for pain management in a patient with a pre-existing SEL shows the safety and feasibility of implanting hardware in the dorsal epidural space in SEL patients [69].

Role of Bariatric Surgery in SEL

There is a paucity of medical literature about the use and efficacy of bariatric surgery in SEL patients [70]. The authors explored the role of bariatric surgery based on the reported one-year mortality of spinal decompression for SEL by Fessler et al. (1992), which appeared to arise from medical comorbidities and resolved with bariatric intervention. Moreover, many of our obese SEL patients had poor compliance with weight loss therapies due to difficulty ambulating, disabling cardiorespiratory issues, or hip and knee osteoarthritis, which resulted in a failed weight loss program. The NIH statement “Gastrointestinal Surgery for Severe Obesity” along with clinical guidelines developed by the National Heart, Lung, and Blood Institute Expert Panel on the identification, evaluation, and treatment of obesity for adults indicate bariatric surgery for patients with a BMI >40 kg/m^2^ or with a BMI >35 kg/m^2^ and one or more significant comorbid conditions. This option is offered when conservative methods of weight loss have been unsuccessful and the patient is at an increased risk for obesity-associated morbidity and mortality.

Bariatric surgery has been reported to produce a long period of sustained weight loss and offered a complete resolution or significant improvement in diabetes mellitus, hyperlipidemia, hypertension, and obstructive sleep apnea [71]. However, the first operative mortality rate for bariatric surgery is approximately 0.2% with up to 25% of patients requiring reoperation within five years. Recovery from bariatric surgery can be delayed by factors such as infection, nutritional deficits, diarrhea, and hemorrhage, among other postoperative complications. This data should be studied considering published mortality and morbidity rates in obese SEL patients who undergo spinal decompression, which is considered a lower-risk spinal procedure. Fessler et al. (1992) reported a 22% mortality rate in SEL patients within a year after decompressive spine surgery, which is likely to be from systemic comorbidities and not the lumbar surgery itself [72]. This suggests that an overall reduction of medical comorbidities by bariatric surgery can offer a reduction in mortality even if a lumbar decompression needs to follow as an additional or delayed intervention.

We present the published data compiling the case reports of SEL that demonstrated clinical and radiological improvement after weight loss programs, diet alterations, and bariatric surgery, including two of our own sleeve gastrectomy cases (Table 2). Improvement in our two obese SEL patients with co-existing mediastinal lipomatosis after sleeve gastric surgery has been presented with MRI evidence of a reduction in thecal sac compression from lipomatosis (Supplementary Image 5).

**Table 2:** Case reports of SEL demonstrating clinical and radiological improvement in BMI either with a weight loss program or sleeve gastrectomy. Two new post-bariatric surgery cases from our series are listed as Case 1 and Case 2 (refer to Figure 5). #van Rooij et al. - 41-year-old male also in Borstlap et al. *Borstlap et al. - No clinical improvement for the 32-year-old male (second case). $ Both Case 1 and Case 2 are presented here for the first time. SEL: spinal epidural lipomatosis

Article	Age	Sex	Symptoms	Intervention	Pre-intervention BMI	Post-intervention BMI
Beges et al. [66]	59	Male	Claudication	Weight loss	36.5	30.1
Beckworth et al. [65]	55	Male	Claudication	Weight loss	31.7	26.7
Kniprath et at. [6]	N/A	Female	Claudication	Weight loss	41.6	25.2
Valcarenghi et al. [70]	48	Male	Claudication	Sleeve gastrectomy	37.4	29.1
^#^van Rooij et al. [67]	41	Male	Right radiculopathy	Weight loss	28.6	22.1
^*^Borstlap et al. [67]	32	Male	Left radiculopathy	Weight loss	29.3	24.1
Borstlap et al. [67]	30	Female	Right radiculopathy	Weight loss	34.0	29.9
Robertson et al. [73]	59	Male	Right radiculopathy	Weight loss	32.9	29.7
Robertson et al. [73]	40	Male	N/A	Weight loss	31.8	26.7
Qasho et al. [10]	44	Male	Left radiculopathy	Weight loss	29.7	26.3
Boutsen et al. [74]	51	Male	Left radiculopathy and claudication	Weight loss	37.6	30.1
Maillot et al. [23]	63	Male	Left radiculopathy and claudication	Weight loss	32.6	25.8
Noël et al. [7]	42	Male	Right radiculopathy	Weight loss	35.5	30.0
Duran et al. [75]	46	Female	Bilateral radiculopathy	Weight loss	32.0	N/A
^$^Case 1	56	Female	Bilateral radiculopathy	Sleeve gastrectomy	49.2	38.4
^$^Case 2	27	Female	Left radiculopathy	Sleeve gastrectomy	62.8	58.6

**Figure 5:**
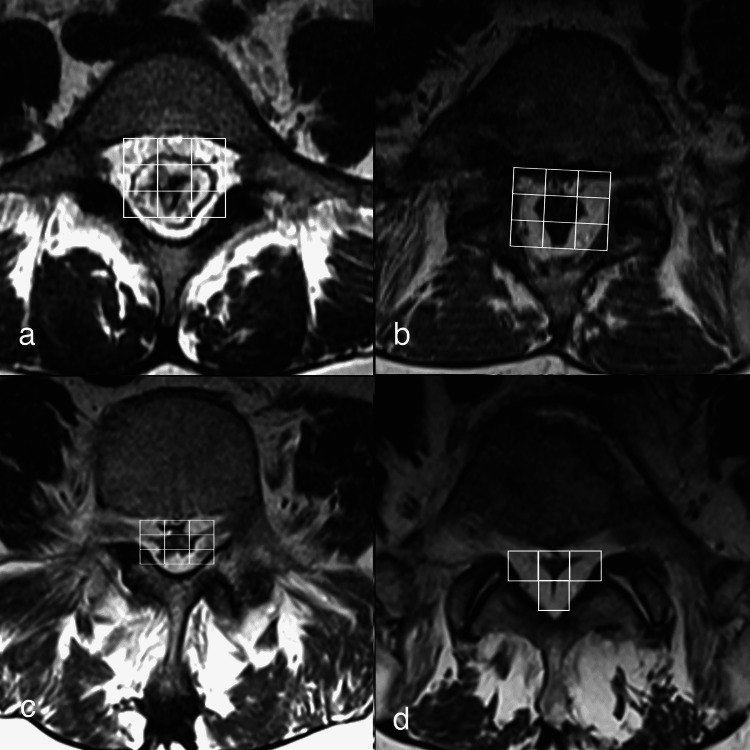
Axial T2-weighted MR images showing some practical applications of superimposed 3x3 grids and a 3+1 grid in certain overlapping grades in the Manjila classification of SEL. (a) shows mild ventral SEL (type 1). (b) shows a central (type 3) thecal sac location with moderate severity, and (c) shows central (type 3) moderate thecal sac compression. In the 3x3 grid technique, the dorsal row of squares shows varying grades of encroachment by hypertrophic facet joints/synovial cyst as shown in a-c. (d) shows severe SEL with the thecal sac restricted only to one small square, with arthritic fluid level within the facet joint, making it a subcategory Alpha (α). The MRI software function of area (cm-square) for exact measurements of the spinal canal and dural sac needs to be compared to the Manjila classification using grids as shown in the images above. SEL: spinal epidural lipomatosis Source: Authors of the current article

Algorithm of Management in Obese and Non-Obese SEL Patients

Patients with SEL typically have lesions, sometimes multiple, in the thoracic or lumbar level with three grades of stenotic severity: mild, moderate, and severe [3]. The more severe stenosis is considered a parameter for surgical intervention (Supplementary Image 6). However, the targeted amount of weight loss as a parameter to consider the failure of conservative management is still debated.

If the patient is non-obese and symptomatic, the mild stenosis can be managed medically with weight loss programs and imaging, with a time-bound follow-up to monitor for worsening on imaging with clinical correlation. If there are co-existing surgical pathologies (e.g., synovial cysts, facetal hypertrophy, disc-osteophyte complex, etc.), a decompressive laminectomy can be advised as deemed necessary based on clinical exam and neurophysiological testing. If there is moderate stenosis, physical therapy and symptomatic management would be the mainstay of treatment and a lumbar laminectomy is offered only if there are co-existing surgical pathologies as mentioned above. If there is severe stenosis in a non-obese patient who is symptomatic, a decompressive laminectomy is the best option to achieve decompression of the affected neural structures.

**Figure 6:**
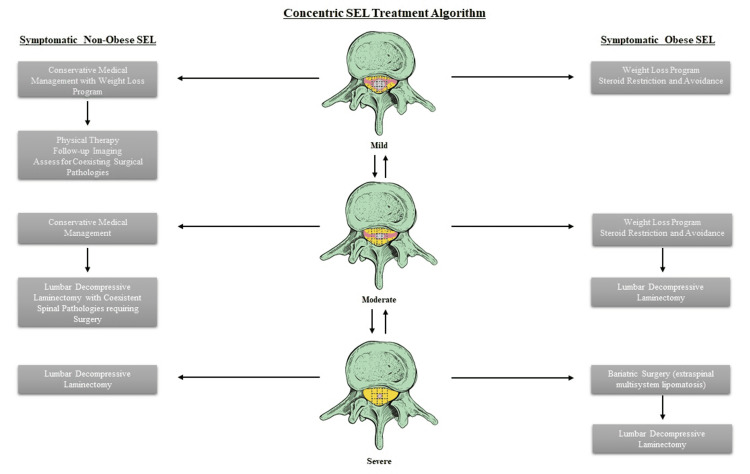
Algorithm for the management of symptomatic SEL in obese and non-obese patients Bariatric surgery is considered for obese patients with symptomatic severe SEL and multisystem lipomatosis, whereas focal lumbar spinal lesions may warrant lumbar surgery (laminectomy/laminoplasty/endoscopic surgery) for decompression. Spinal decompression is also offered to symptomatic SEL patients with co-existing spondylotic pathologies like facet hypertrophy, disc osteophyte, or a synovial cyst. SEL: spinal epidural lipomatosis Source: Authors of the current article

In symptomatic obese patients, mild cases can be managed with weight loss and conservative medical management, including steroid avoidance or restriction. Moderate stenosis can be treated with the same modalities except for the consideration of focal spinal decompression with laminectomy if there are co-existing surgical pathologies. In severe cases, particularly those associated with extraspinal lipomatosis, bariatric surgery can offer a widely systemic clinic-radiological improvement. A lumbar decompressive surgery can then be offered if there are co-existing pathologies that contribute to non-lipomatous canal stenosis (Figure 6).

## Conclusions

Patients with SEL can present with neurogenic claudication, radiculopathy, myelopathy, and, in rare cases, cauda equina syndrome. We present the role of extraspinal lipomatosis (mediastinal or visceral) and its effect on SEL for the first time in literature and propose a simple and user-friendly radiologic grading system of the three classic SEL locations (ventral, dorsal, and concentric) that range from mild to severe stenosis. This grading system can be performed and interpreted by the surgeon or radiologist with a simple 3x3 grid (or 3+1 at the L5-S1 level) manually drawn on axial spinal imaging, even in those with associated canal stenosis or scoliosis. A management algorithm for SEL is proposed for treating obese and non-obese patients with a given role for bariatric surgery in the former, especially when there is associated significant extraspinal lipomatosis. We propose that SEL patients with focal spinal surgical pathologies (e.g., synovial cysts, facet hypertrophy, disc-osteophyte complex, etc.) should be offered a decompressive laminectomy as the first line of management. A randomized controlled trial of a large sample population is necessary to define the optimum treatment option for SEL and patient-reported clinical outcome after bariatric surgery in symptomatic obese patients with SEL, particularly in severe Manjila Type III cases.

## Figures and Tables

**Figure 7:**
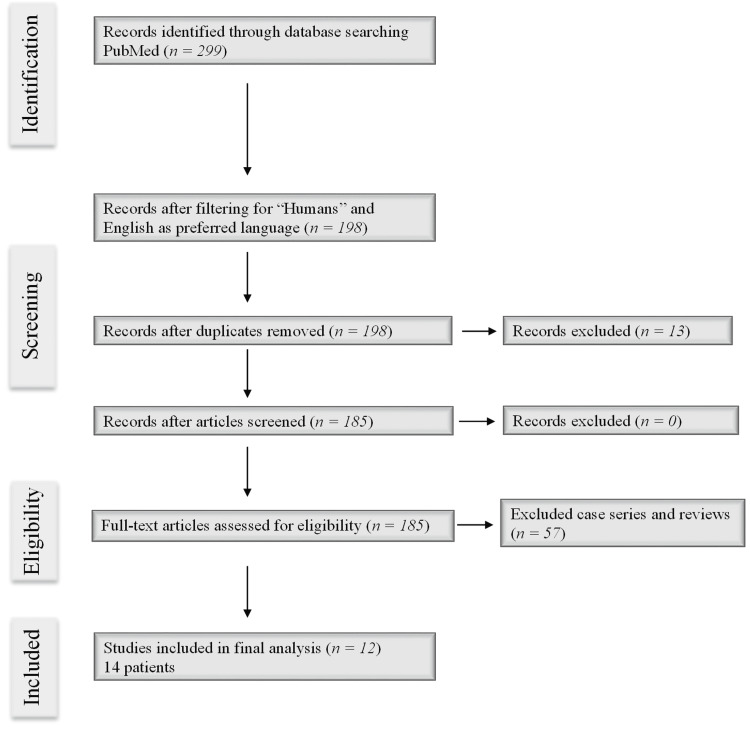
Supplementary Image 1 PRISMA flowchart search strategy of current literature and article selection for analysis PRISMA: Preferred Reporting Items for Systematic Reviews and Meta-Analyses Source: Authors of the current article

**Figure 8:**
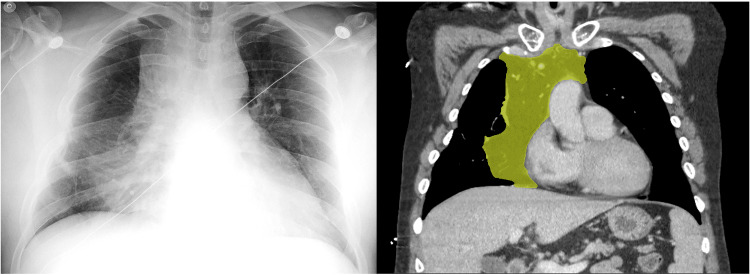
Supplementary Image 2 Anteroposterior X-ray (left) and post-contrast coronal CT (right) of a 38-year-old male with mediastinal lipomatosis (yellow shaded area) Source: Authors of the current article

**Figure 9:**
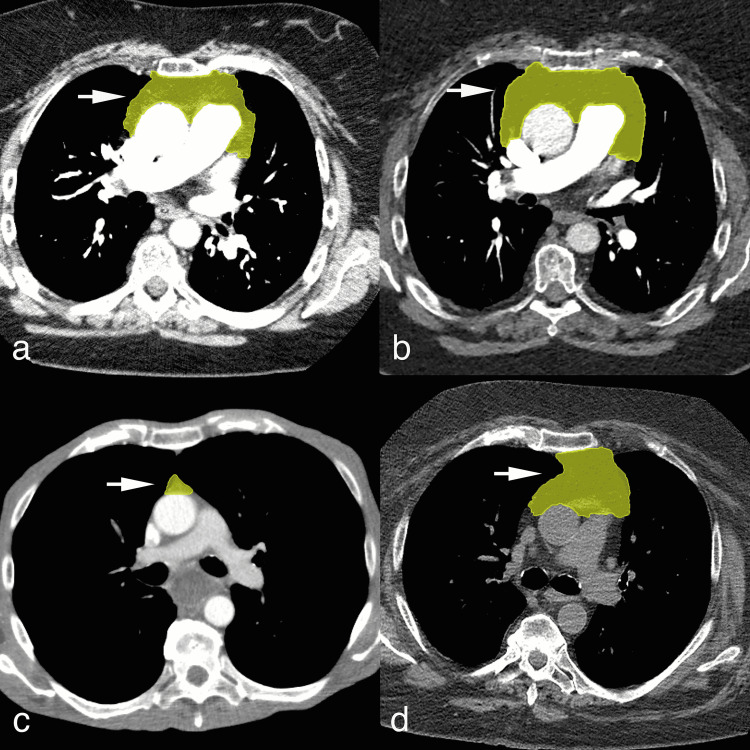
Supplementary Image 3 Mediastinal lipomatosis: radiological progression with increasing BMI Upper row: Post-contrast axial CTs of a 64-year-old female with mediastinal lipomatosis (yellow shaded area) that progressed with an increase in BMI from a) 32.0 to b) 37.7 over a period of 7.5 years despite having a prior vertical band gastroplasty. Lower row: Post-contrast and non-contrast axial CTs of a 68-year-old female with mediastinal lipomatosis that progressed with an increase in BMI from c) 21.9 to d) 43.2 over a period of 6.5 years after having a prior sleeve gastrectomy. BMI: body mass index Source: Authors of the current article

**Figure 10:**
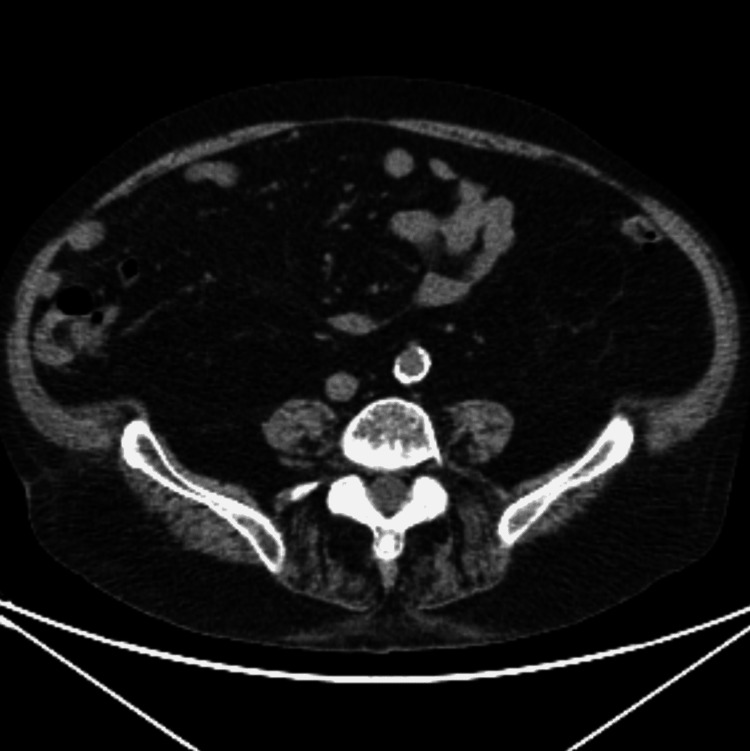
Supplementary Image 4 Axial non-contrast CT demonstrating extensive extraspinal (intra-abdominal) lipomatosis with minimal subcutaneous lipomatosis Source: Authors of the current article

**Figure 11:**
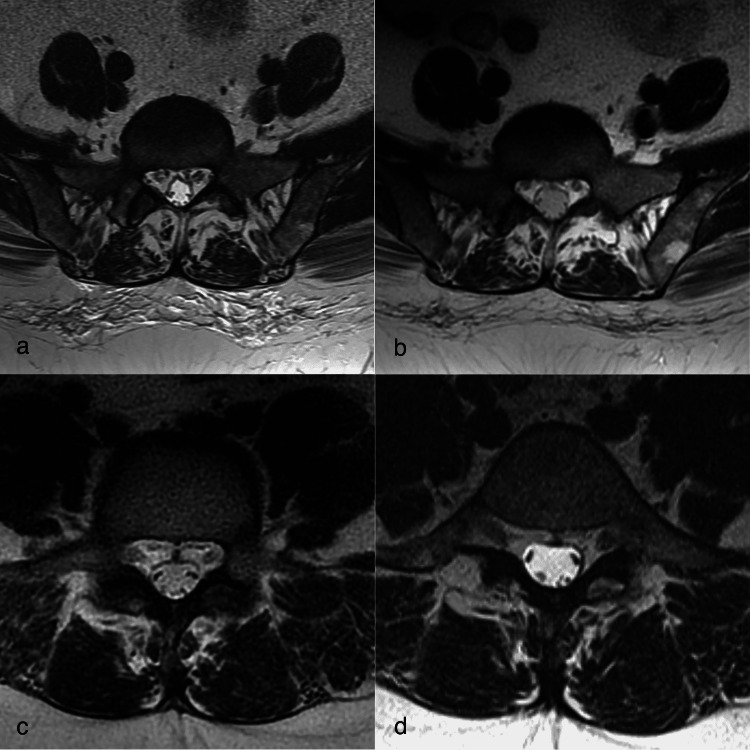
Supplementary Image 5 Upper row: axial T2-weighted MR images at L5-S1 in a 56-year-old female with a) an initial BMI of 49.2, b) a BMI of 38.4 after an elective sleeve gastrectomy. Lower row: Axial T2-weighted MR images in a 27-year-old female with a c) BMI of 62.8, and d) a BMI of 58.6 after an elective sleeve gastrectomy. Note the midline septum or midline lumbosacral meningovertebral ligament dividing the anterior epidural space into two compartments. BMI: body mass index Source: Authors of the current article

**Figure 12:**
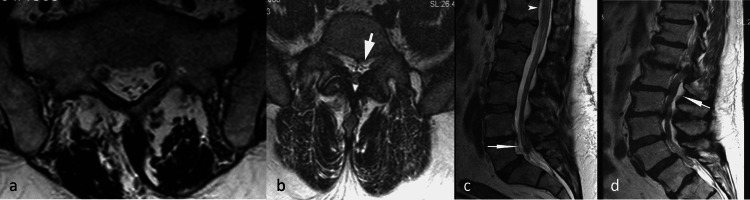
Supplementary Image 6 a and b: Axial T2-weighted MR images of the lumbosacral spine showing a) severe Manjila type I (ventral) SEL; and b) severe SEL with facet arthropathy. c and d: Sagittal T2-weighted MR images showing c) upper arrowhead (normal thecal sac) and long white arrow showing hypoplastic L5 body, Type I A-SEL. d) shows the effect of upper lumbar canal stenosis (upper white arrow) and hypoplastic L5 body. SEL: spinal epidural lipomatosis Source: Authors of the current article
